# Induction heating combined with antibiotics or SAAP-148 effectively reduces biofilm-embedded *Staphylococcus aureus* on a fracture-related implant mimic

**DOI:** 10.1302/2046-3758.145.BJR-2024-0341.R1

**Published:** 2025-05-20

**Authors:** Marielle Verheul, Anne A. Wagenmakers, Rob G. H. H. Nelissen, Peter H. Nibbering, Bart G. Pijls

**Affiliations:** 1 Department of Orthopedics, Leiden University Center for Infectious Diseases (LUCID), Leiden University Medical Center, Leiden, Netherlands; 2 Leiden University Center for Infectious Diseases (LUCID), Leiden University Medical Center, Leiden, Netherlands; 3 Department of Orthopedics, Leiden University Medical Center, Leiden, Netherlands

**Keywords:** NCIH, Bacteriophage ISP, Antimicrobial peptide, Rifampicin/ciprofloxacin, FRI, Staphylococcus aureus, Antibiotics, Biofilms, Rifampicin, bacteria, MRSA, peptide, staining, methicillin-sensitive S. aureus, infections

## Abstract

**Aims:**

Fracture-related infections and the associated treatment failure burden our society and healthcare system significantly. As an alternative approach, we investigated the effect of non-contact induction heating (NCIH) against *Staphylococcus aureus* within mature biofilms. In addition, we assessed the ability of antibiotics, the antimicrobial peptide SAAP-148, and bacteriophage (phage) ISP to enhance the efficacy of NCIH, thereby allowing the use of lower temperatures during NCIH.

**Methods:**

Clinical isolates of methicillin-resistant and methicillin-sensitive *S. aureus* (methicillin-resistant *S. aureus* (MRSA), methicillin-sensitive *S. aureus* (MSSA)) were cultured for seven days on Ti-6Al-7Nb (mimicking fracture plates) discs to obtain mature biofilms. Biofilms were exposed to 60°C to 80°C NCIH. In addition, biofilms were sequentially exposed to 60°C to 70°C NCIH and rifampicin/ciprofloxacin, SAAP-148, or phage ISP. Biofilm-embedded bacteria were harvested by sonication to determine the bacterial load and visualized by confocal microscopy (LIVE/DEAD).

**Results:**

NCIH to 60°C, 70°C, and 80°C reduced biofilm-embedded MRSA and MSSA by 2.3-log, 4.9-log, 5.5-log, and 1.1-log, 3.4-log, and 6.6-log CFU/ml, respectively. LIVE/DEAD staining revealed NCIH-induced bacterial cell death throughout the biofilm layers. The sequential combination of rifampicin/ciprofloxacin at 10 µg/ml and 1,280 µg/ml (MRSA) or 156 µg/l and 64 µg/ml (MSSA) and 70°C NCIH synergistically reduced biofilm-embedded bacteria by 2.7-log and 3.7-log CFU/ml, respectively, while the alternating exposure order reduced bacterial counts by -0.1 and 1.7-log CFU/ml. SAAP-148 at 51.2 µM followed by 70°C NCIH further diminished biofilm-embedded MRSA and MSSA by 2.3-log and 1.5-log CFU/ml, respectively. No significant reductions were observed for NCIH combined with phage ISP compared to these treatments alone.

**Conclusion:**

NCIH effectively reduced biofilm-embedded *S. aureus* on Ti-6Al-7Nb in a heat-dependent fashion. Rifampicin/ciprofloxacin and SAAP-148, but not phage ISP, enhanced the efficacy of NCIH. Antibiotic exposure at suboptimal concentrations followed by NCIH was more effective than vice versa, suggesting that the application of this approach might be most suitable in clinical situations where antibiotic treatment has already started.

Cite this article: *Bone Joint Res* 2025;14(5):485–494.

## Article focus

- Clinical isolates of *Staphylococcus aureus* within seven-day mature biofilms on a metal implant mimic were exposed to non-contact induction heating (NCIH) ranging from 60°C to 80°C.
- Biofilm-embedded *S. aureus* was sequentially exposed to NCIH and rifampicin/ciprofloxacin, the antimicrobial peptide SAAP-148, or bacteriophage ISP to enhance treatment efficacy and allow the use of lower NCIH temperatures.

## Key messages

- NCIH ranging from 60°C to 80°C effectively eliminated clinical isolates of *S. aureus* within mature biofilms in a heat-dependent fashion.
- The sequential combination of NCIH with antibiotics or the antimicrobial peptide SAAP-148, but not bacteriophage ISP, effectively reduced biofilm-embedded *S. aureus* and allowed the use of lower NCIH temperatures.
- Antibiotics exposure at suboptimal concentrations followed by NCIH was more effective than vice versa, indicating that this approach might be most suitable in clinical situations where antibiotic treatment has already started.

## Strengths and limitations

- We incorporated clinical *S. aureus* isolates, a commonly used fracture-related metal implant disc, and a seven-day mature biofilm model to study the efficacy of our combinatory approaches.
- We determined the effects of antibiotic-based and completely non-antibiotic-based approaches to fight *S. aureus* within mature biofilms on an implant mimic.
- Clinically relevant factors such as the host immune system were not integrated in this model.

## Introduction

Implant-related infections, such as fracture-related infections (FRIs) and periprosthetic joint infections (PJIs), are difficult to treat. The current treatment involves surgical debridement or revision surgery followed by extensive antibiotic use, which is often ineffective due to bacterial biofilm formation on the metal implant surface.^1,2^*Staphylococcus aureus* is commonly associated with these biofilm-associated infections and treatment failure.^3,4^ The biofilm matrix hampers the penetration and efficacy of antibiotics, and biofilm-embedded bacteria can be 10 to 1,000 times more tolerant of antibiotics than planktonic bacteria. That is, biofilms can harbour a variety of heterogeneous subpopulations with different antibiotic susceptibilities. Regions within the biofilm with limited nutrients and hypoxia give rise to populations with lower metabolic activity, such as persisters, which are highly antibiotic-tolerant.^5^ These characteristics and other dynamic processes within the biofilm contribute to bacterial survival and persistence of these infections. This could lead to the selection of more antibiotic-resistant strains, adding to the global problem of antimicrobial resistance (AMR).^6,7^

Therefore, alternative strategies to combat biofilm-associated infections are required. Non-contact induction heating (NCIH) forms an interesting approach to eradicate (biofilm-embedded) bacteria attached to metal implants. Using electromagnetic fields, NCIH generates eddy currents in the metal implant, resulting in heating of the implant surface that can effectively eliminate biofilm-embedded bacteria.^8-10^ NCIH can be applied invasively during surgery by using heat to eliminate bacteria in inaccessible areas for surgical debridement while keeping the soft-tissues away to limit tissue damage. If surgical treatment is not possible or recommended, NCIH could be applied as a non-invasive approach by heating the implant through the skin. In that case, homogeneous heating of the implant and temperature control are even more crucial to control tissue damage.

Importantly, NCIH could complement antibiotics to fight biofilm-associated infections, allowing lower NCIH temperatures and/or antibiotic dosing. Previous studies have shown that NCIH can synergize with antibiotics in reducing *Pseudomonas aeruginosa, Staphylococcus epidermidis*, and methicillin-sensitive *S. aureus* (MSSA) biofilms.^11-14^ Unlike antibiotics, NCIH eradicated methicillin-resistant *S. aureus* (MRSA) persisters within biofilms, illustrating that this physical approach can target bacteria independent of their metabolic activity.^15^

The efficacy of NCIH at lower temperatures could also be improved by non-antibiotic approaches, such as the synthetic and antibiofilm peptide (SAAP)-148, which has been shown to eliminate drug-resistant (biofilm-embedded) bacteria, including persisters.^16,17^ Previously, NCIH combined with SAAP-148 slightly improved the reduction of persisters within antibiotics-exposed mature MRSA biofilms.^15^ Alternatively, NCIH could be combined with lytic bacteriophages (phages), viruses that can specifically infect and eliminate bacteria of a particular (strain within) species. Regarding the efficacy of phages, previous work has demonstrated that phage ISP as a single approach could not effectively reduce mature biofilm-embedded *S. aureus* in vitro*.*^18^ Whether the efficacy of NCIH at different temperatures can be enhanced by SAAP-148 or phage ISP exposure to *S. aureus* biofilms remains undetermined. Sequential exposure to these different methods would be preferable to simultaneous exposure since NCIH could affect the stability and efficacy of antibiotics, SAAP-148, and phage ISP in the latter. It remains unclear whether the order of exposure to NCIH and antimicrobials affects the efficacy of the sequential treatment combinations, which could be essential information for future applications of these methods.

In this study, we investigated the efficacy of NCIH on MSSA and MRSA clinical isolates within mature biofilms on a commonly used fracture-related implant material (Ti-6Al-7Nb). Moreover, we assessed whether the sequential combination of antibiotics, SAAP-148, and phage ISP with NCIH could enhance the efficacy of NCIH at lower temperatures. The latter may cause fewer adverse events to the surrounding tissue of the infected metal implant.

## Methods

### Metal implant mimic

Medical grade titanium-6% aluminium-7% niobium (Ti-6Al-7Nb) (TAN; iso5832/11 discs) was used as a fracture-related implant mimic.^19^ Ti-6Al-7Nb discs were handled as described previously,^15^ i.e. overnight submersion in 70% EtOH, air drying, and subsequent sterilization by autoclavation after usage.

### Bacterial strains

The current study used a methicillin-sensitive *S. aureus* (MSSA; coded as LUH15393) clinical isolate from a patient with PJI and a methicillin-resistant *S. aureus* (MRSA, coded as LUH14616 (sequence type 247, NCCB 100829; kindly provided by Dr S. Croes (Department of Medical Microbiology, Maastricht University Medical Center, Maastricht, Netherlands))). Bacterial strains were frozen in 20% glycerol and stored at -80°C until use.

### Biofilm culture

Bacteria were spread from frozen stocks on trypticase soy agar plates with 5% sheep blood (43009, Biomerieux) and incubated overnight at 37°C. Single colonies were taken from this plate and cultured in tryptone soy broth (TSB, CM0129, Oxoid) for 2.5 hours (200 rpm, 37°C) to reach a mid-logarithmic phase. Bacteria were harvested by centrifugation (1,000 ×g for 10 mins) and washed with phosphate-buffered saline (PBS, pH 7.4). Based on the optical density (600 nm), bacteria were diluted to 1 × 10^7^ CFU/ml in brain-heart infusion broth (BHI, CM1135, Oxoid) and 100 µl of this suspension was added into 96-well flat-bottom polystyrene plates containing Ti-6Al-7Nb discs. The plates were sealed with a breathable rayon sealing film (391 to 1262, VWR) and incubated in a humidified environment at 37°C for seven days to allow mature biofilm formation. Medium controls and inoculum were checked to monitor possible contamination.

### Antimicrobial agents

Antibiotics: Rifampicin (13292-46-1, Sigma Aldrich, USA) was diluted in DMSO (4 g/l) and ciprofloxacin (86393-32-0, Sigma Aldrich) in MilliQ water (25.6 g/l). All antibiotics were stored at -20°C until use. The minimal bactericidal concentration (MBC), corresponding to 99.9% eradication of bacteria,^20^ of rifampicin and ciprofloxacin was determined by broth microdilution following EUCAST guidelines.^21^ Thereafter, ten-fold bacterial dilutions were plated on Mueller Hinton agar (CM0337, Oxoid) plates for microbiological assessment (colony forming units (CFU)/ml) after overnight incubation at 37°C. Biofilm-embedded LUH15393 (MSSA) was exposed to rifampicin and ciprofloxacin at 15.6 µg/l and 6.4 µg/ml, 78 µg/l and 32 µg/ml, and 156 µg/l and 64 µg/ml, respectively. LUH14616 (MRSA), which is rifampicin- and ciprofloxacin-resistant, was exposed to rifampicin and ciprofloxacin at 1 and 128 µg/ml, 5 and 640 µg/ml, and 10 and 1,280 µg/ml, respectively. Alternatively, both strains were exposed to rifampicin and ciprofloxacin at 4 µg/ml.

Synthetic antimicrobial and antibiofilm peptide (SAAP)-148: SAAP-148 was kindly provided by R.A. Cordfunke (Department of Immunology, Peptide Immunology, Leiden University Medical Center, Leiden, Netherlands). Solid phase chemistry using an automated multiple peptide synthesizer (SyroII; MultiSyntech, Germany) was used to synthesize SAAP-148, as described previously.^16^ Mass spectrometry was used to confirm the molecular mass of the N-terminal acetylated and C-terminal amidated peptide. Reverse-phase high-performance liquid chromatography and detection at 214 nm showed that the purity of the peptide exceeded 95%. SAAP-148 was lyophilized and stored at -20°C until use.

Bacteriophage ISP: bacteriophage (phage) ISP (dsDNA, non-segmented, NC_047720^22^) was freshly propagated using the double overlay method with MRSA (LUH14616) as described previously.^18^ The sensitivity of the clinical isolates used here to phage ISP was confirmed earlier.^18^ Phage stocks were stored at 4°C until use.

### Non-contact induction heating

Bacterial biofilms on Ti-6Al-7Nb discs were exposed to a pulsed electromagnetic field (PEMF) with our custom-built induction heater (max power 135 Watts, frequency 101 kHz) as described previously.^15^ Briefly, Ti-6Al-7Nb discs were put in a 2 ml micro-tube with 100 µl PBS, which was inserted in the 50 ml plastic centrifuge tube with a silicon mould. This tube was consistently put in the same position in the coil of the induction heater to control the temperature settings for every single disc. That is, non-contact induction heating (NCIH) for two, four, and six minutes corresponded to reaching the target temperature of approximately 60°C, 70°C, and 80°C on the surface of the Ti-6Al-7Nb disc.^15^ After NCIH exposure, the temperature of the discs dropped to 37°C within 30 seconds.

### Biofilm exposure to induction heating and/or antimicrobial agents

Biofilms on Ti-6Al-7Nb discs were exposed to NCIH (60°C, 70°C, or 80°C), rifampicin/ciprofloxacin, SAAP-148, or phage ISP, and to sequential combinations of NCIH with rifampicin/ciprofloxacin, SAAP-148, or phage ISP. Before and after each exposure, biofilms were washed twice with PBS to remove non-biofilm-associated bacteria. Biofilms on Ti-6Al-7Nb discs were transferred to a fresh 96-well flat-bottom polystyrene plate with 100 µl of rifampicin/ciprofloxacin at concentrations mentioned above in BHI, or phage ISP at 10^8^ plaque-forming units (PFU)/ml in BHI, and exposed at 37°C for 24 hours. Alternatively, biofilms on Ti-6Al-7Nb discs were transferred to 96-well flat-bottom polypropylene plates with 100 µl of SAAP-148 at 12.8, 25.6, or 51.2 µM in PBS and exposed at 37°C for two hours. To ensure that the metal implant cooled down to 37°C after exposure to NCIH, biofilms on Ti-6Al-7Nb discs were transferred several minutes after NCIH into the 96-well flat-bottom plate containing rifampicin/ciprofloxacin, SAAP-148, or phage ISP in the desired medium. The treatment duration with these antimicrobial agents was similar for both sequential treatment orders. Plates were sealed with a non-breathable plastic sealing film (WB 2-3830, Westburg Life Sciences, Netherlands). BHI or PBS served as controls. After the desired (combined) antimicrobial approach, bacteria were sonicated from the biofilm in the presence of PBS, and microbiologically assessed as described for the MBC. Phage-exposed biofilms were sonicated in 0.9% saline supplemented with 10% ammonium iron (II) sulphate hexahydrate (FAS, 100 mM; F1543, Sigma Aldrich) to neutralize the residual activity of extracellular phages.^23^ Medium controls were included to determine possible contamination.

### Confocal laser scanning microscopy

Viable- and non-viable bacteria within mature MRSA biofilms on Ti-6Al-7Nb discs were visualized using LIVE/DEAD staining, i.e. SYTO-9 green (5 mM, S34854; Invitrogen, Thermo Fisher Scientific, USA) and TO-PRO-3-iodide (1 mM, T3605, Invitrogen) red fluorescent nucleic acid-binding dyes, respectively. Biofilms were washed twice with 100 µl 0.9% saline before staining with 100 µl SYTO-9 at 3 µM in 0.9% saline for 30 minutes at room temperature in the dark. Next, biofilms were washed once with 100 µl 0.9% saline and counterstained with 100 µl TO-PRO-3-iodide 4 µM in 0.9% saline for 15 minutes at room temperature in the dark. Biofilms were washed once with 100 µl 0.9% saline, and Ti-6Al-7Nb discs with biofilms were carefully transferred to µ-slide eight-well glass bottom chambered coverslip (80827, Ibidi) with 250 µl 0.9% saline. SYTO-9 and TO-PRO-3-iodide were visualized using 488 nm Ar^+^ laser line (emission collected at 500 to 590 nm) and the 633 nm He-Ne laser line (emission collected at 645 to 795 nm), respectively, using Andor Dragonfly 500 (Oxford Instruments, UK). Vertical representation of biofilms on Ti-6Al-7Nb discs was made with z-stacks (63× magnification), and images were analyzed with Imaris Software (version 9.5.1). Images were selected based on their representation of the trend observed in three independent experiments.

### Statistical analysis

The mean log CFU/ml with 95% CI was determined after log-transforming the CFU/ml data as previously recommended, using GraphPad Prism 9.3.1 (GraphPad Software, USA). After that, the mean log CFU difference (Δ mean) with corresponding 95% CI was calculated as described previously,^18,24^ comparing the effect of sequential exposures to the effect of the most effective of the two single approaches. Additive reductions were classified as mean differences ≥ 1 and < 2 log CFU/ml, whereas synergistic reductions amounted to mean differences ≥ 2 log CFU/ml.^25,26^

## Results

### Effect of NCIH on biofilm-embedded *S. aureus*

**Fig. 1:**
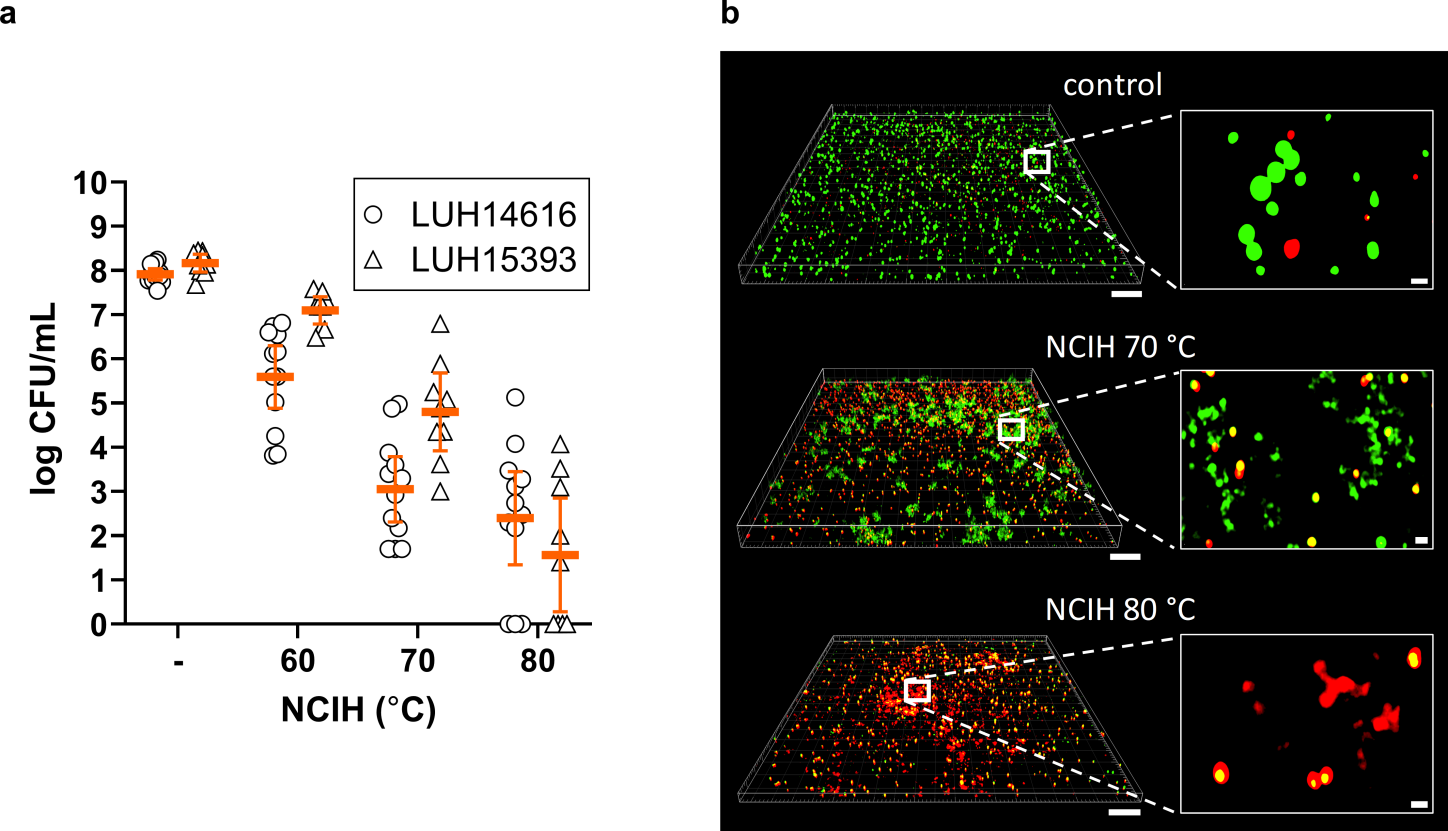
Effect of non-contact induction heating (NCIH) on mature biofilm-embedded *Staphylococcus aureus* on a metal implant mimic. a) Mature biofilms of methicillin-resistant *S. aureus* (MRSA; LUH14616) and methicillin-sensitive *S. aureus* (MSSA; LUH15393) on titanium-6% aluminium-7% niobium (Ti-6Al-7Nb) discs were exposed to NCIH for two, four, or six minutes, corresponding to 60°C, 70°C, and 80°C, respectively. Then, biofilms on Ti-6Al-7Nb discs were sonicated and bacterial counts were determined microbiologically (colony-forming units (CFU)/ml). The solid line indicates the mean CFU/ml with the corresponding 95% CIs of the log-transformed data. Results are from three independent experiments in triplicate or quadruplicate. b) Mature MRSA biofilms on Ti-6Al-7Nb discs were exposed to 70°C or 80°C NCIH, and biofilm-embedded bacteria were visualized by LIVE (green)/DEAD (red) staining using confocal microscopy. The apical biofilm is depicted on the bottom, and the basal biofilm is depicted on top of the z-stack. The bars of the z-stacks and zoom-ins represent 20 μm and 1 μm, respectively.

First, we determined the effect of NCIH on established mature biofilms of MRSA and MSSA on a fracture-related metal implant mimic. Our results showed that bacterial counts within mature *S. aureus* biofilms decreased in response to increasing temperatures on the implant surface. That is, NCIH to 60°C, 70°C, and 80°C reduced biofilm-embedded *S. aureus* by a mean 2.3-log, 4.9-log, and 5.5-log CFU/ml, and 1.1-log, 3.4-log, and 6.6-log CFU/ml, respectively (Figure 1a, Supplementary Table i). The bacterial load in the peri-implant fluid of induction-heated biofilms was comparable to or lower than that within biofilms, indicating that this antibiofilm effect was not due to the dislodgement of viable bacteria in the fluid surrounding the implant (Supplementary Figure a). To investigate the distribution of bacterial cell death throughout the biofilm after NCIH, MRSA within mature biofilms on Ti-6Al-7Nb discs was exposed to 70°C or 80°C NCIH and subsequently visualized using LIVE/DEAD staining. Bacterial cell death was observed throughout the various layers of the heat-exposed biofilm (Figure 1b). Of note, biofilms exposed to NCIH showed clustering of staining at the basal layer, in which some viable bacteria could be observed. Altogether, induction heating effectively eliminated biofilm-embedded *S. aureus* on the metal implant mimic.

### Effect of NCIH combined with rifampicin/ciprofloxacin on biofilm-embedded *S. aureus*

**Fig. 2:**
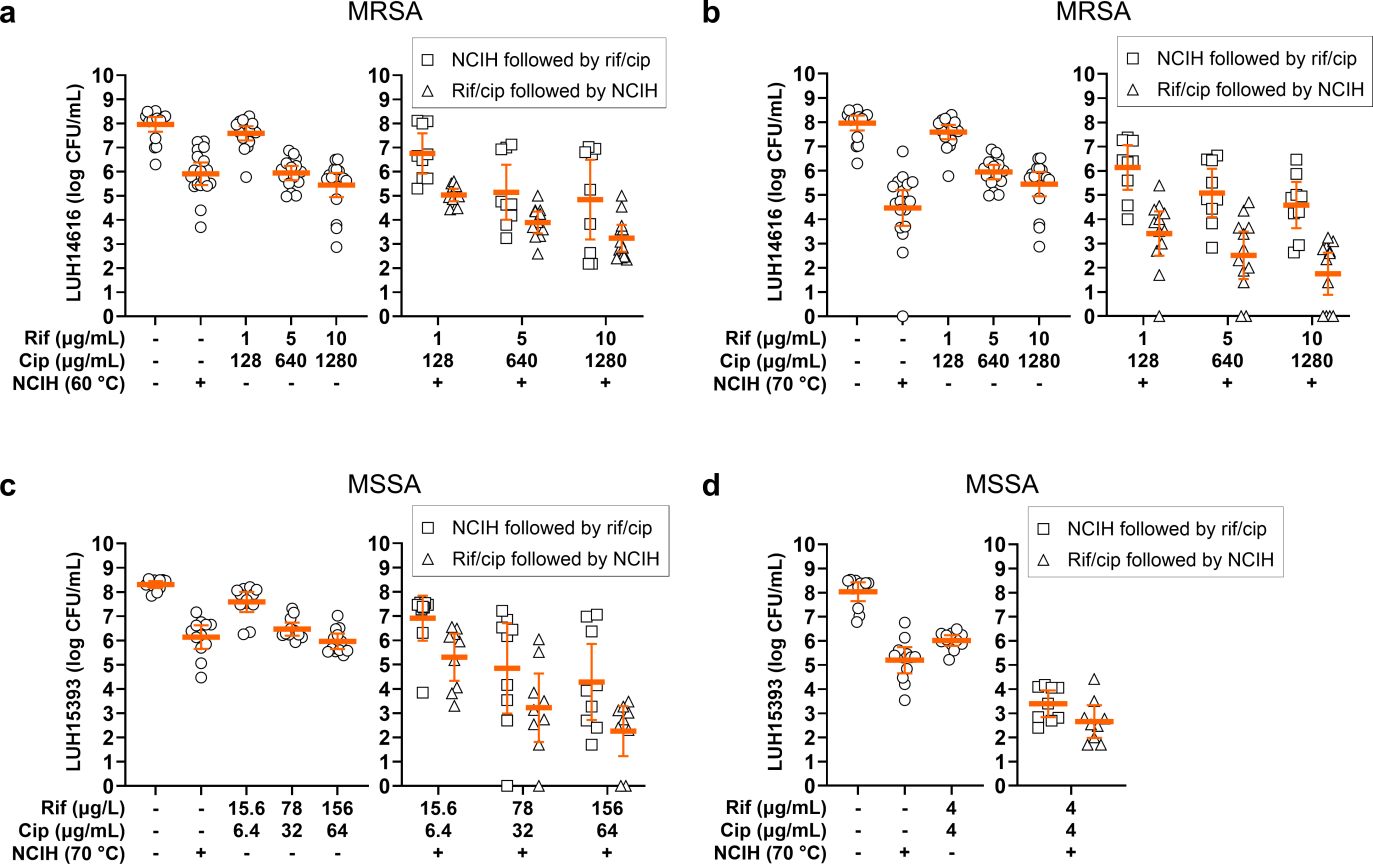
Effect of sequential exposure to rifampicin/ciprofloxacin and non-contact induction heating (NCIH) on *Staphylococcus aureus* biofilms. Mature biofilms of a) and b) methicillin-resistant *S. aureus* (MRSA) and c) and d) methicillin-sensitive *S. aureus* (MSSA) on titanium-6% aluminium-7% niobium (Ti-6Al-7Nb) discs were sequentially exposed to NCIH to a) 60°C or b), c), and d) 70°C and rifampicin/ciprofloxacin (rif/cip) for 24 hours in brain-heart infusion broth (BHI), or vice versa. The unexposed controls were incubated in BHI. Bacteria were harvested from biofilms by sonication in phosphate-buffered saline, and bacterial counts were determined microbiologically (colony-forming units (CFU)/ml). The solid line indicates the mean CFU/ml with the corresponding 95% CI of the log-transformed data. Results are from three independent experiments in triplicate or quadruplicate.

Second, we investigated the potential of rifampicin/ciprofloxacin to improve the efficacy of NCIH against biofilm-embedded *S. aureus*, allowing the use of NCIH at lower temperatures (60°C to 70°C). As simultaneous exposure to 60°C and 70°C NCIH and antibiotics could affect the antibiotic’s efficacy, biofilm-embedded *S. aureus* was sequentially exposed to antibiotics and NCIH. MRSA (rifampicin/ciprofloxacin-resistant) within mature biofilms was used as proof of concept, illustrating that exposure to 60°C or 70°C NCIH and rifampicin/ciprofloxacin additionally reduced biofilm-embedded bacterial counts (Figures 2a and 2b, Supplementary Table ii). Notably, exposure to the antibiotics at the highest tested concentration before exposure to 70°C NCIH effectively reduced bacterial counts by an additional mean 2.7-log CFU/ml. Similar synergistic reductions were observed by this combination when applying clinically reachable concentrations of rifampicin/ciprofloxacin (4 µg/ml) (Supplementary Figure b). In contrast, no additional reductions were observed for the alternative treatment order (Figure 2b, Supplementary Table ii, Supplementary Figure b).

Next, biofilm-embedded MSSA (rifampicin/ciprofloxacin-sensitive) were exposed to 70°C NCIH, followed by rifampicin/ciprofloxacin, or vice versa. Exposure to the antibiotics at the two highest tested concentrations prior to 70°C NCIH synergistically reduced bacterial counts by a mean 2.9-log and 3.7-log CFU/ml, respectively, whereas the other treatment order additively reduced bacterial counts by a mean 1.3-log and 1.7-log CFU/ml (Figure 2c). As this suggests that the biofilm-embedded bacteria proliferate in the presence of the antibiotics following exposure to NCIH, we investigated whether this was due to insufficient antibiotic suppression. Hence, the NCIH-treated MSSA biofilms were exposed to the antibiotics at 4 µg/ml, an extremely high rifampicin concentration (256 × the MBC). As a result, bacterial counts were more similarly reduced upon antibiotic exposure followed by 70°C NCIH and vice versa, i.e. mean 2.5-log and 1.8-log CFU/ml, respectively (Figure 2d, Supplementary Table iii). Thus, rifampicin/ciprofloxacin combined with NCIH, especially to 70°C, further reduced biofilm-embedded *S. aureus*. Exposure to NCIH prior to antibiotics was less effective than the other way around, and this was related to the applied antibiotic concentration and bacterial sensitivity to the antibiotics.

### Effect of NCIH combined with SAAP-148 on biofilm-embedded *S. aureus*

**Fig. 3:**
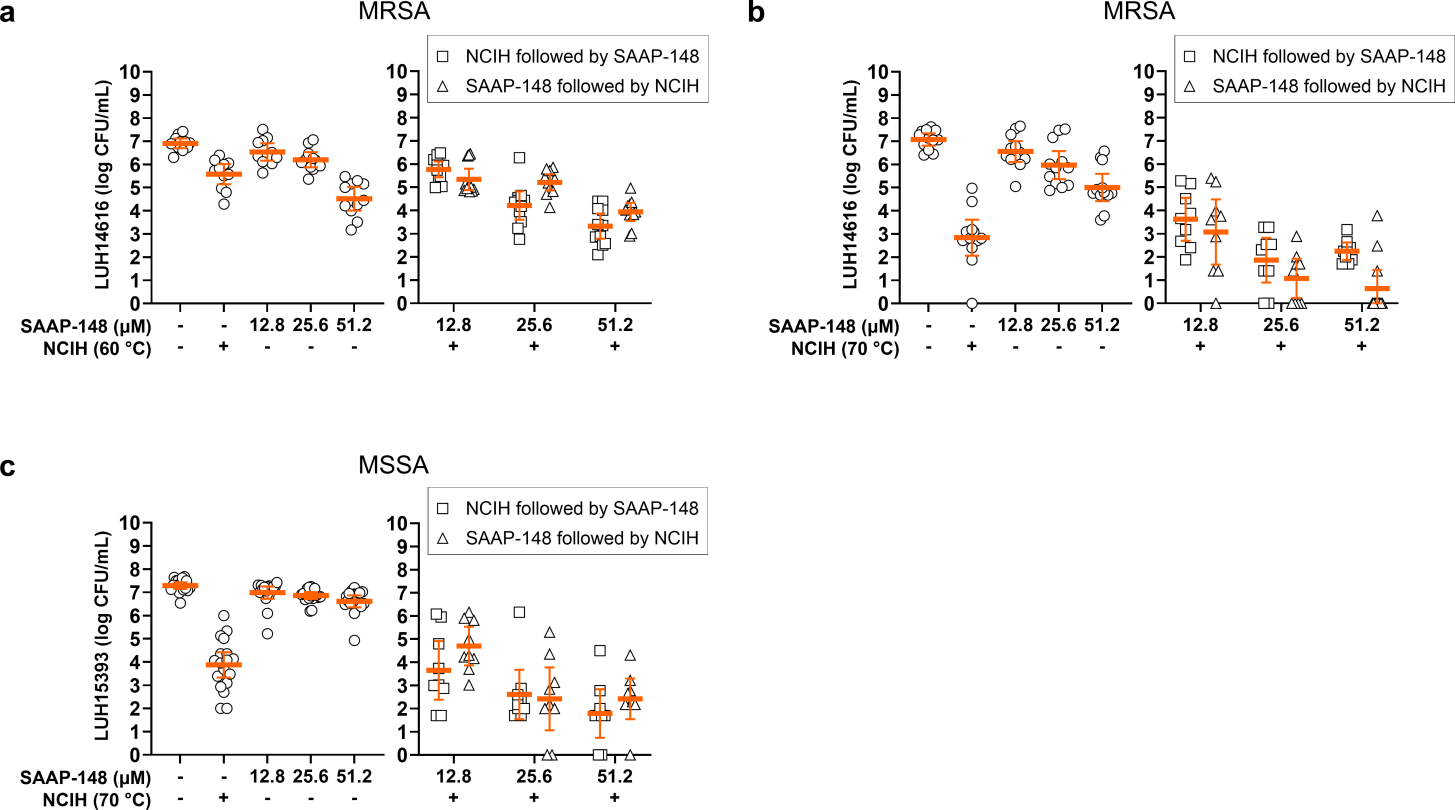
Effect of sequential exposure of *Staphylococcus aureus* biofilms to the synthetic antimicrobial and antibiofilm peptide (SAAP)-148 and non-contact induction heating (NCIH). Mature biofilms of a) and b) methicillin-resistant *S. aureus* (MRSA) and c) methicillin-sensitive *S. aureus* (MSSA) on titanium-6% aluminium-7% niobium (Ti-6Al-7Nb) discs were sequentially exposed to a) 60°C or b) and c) 70°C NCIH and SAAP-148 for two hours in phosphate-buffered saline (PBS), or vice versa. The unexposed controls were incubated in PBS. Bacteria were harvested from biofilms by sonication in PBS, and bacterial counts were determined microbiologically (colony-forming units (CFU)/ml). The solid line indicates the mean CFU/ml with the corresponding 95% CIs of the log-transformed data. Results are from three or four independent experiments in triplicate or quadruplicate.

Next, we determined the ability of the antimicrobial peptide SAAP-148 to enhance the efficacy of NCIH when sequentially exposed to mature biofilm-embedded *S. aureus*. The results showed that NCIH to 60°C prior to SAAP-148 (25.6 and 51.2 µM) exposure, but not the other way around, additionally reduced MRSA counts (Figure 3a, Supplementary Table iv). In addition, SAAP-148 at 51.2 µM followed by 70°C NCIH reduced biofilm-embedded MRSA by a mean 2.3-log CFU/ml (Figure 3b, Supplementary Table iv), while this effect was not observed for the alternating exposure order. Thereafter, we determined the effect of sequential exposure to SAAP-148 and 70°C NCIH on biofilm-embedded MSSA. Bacterial counts were additionally reduced by exposure to SAAP-148 at 25.6 µM and 70°C NCIH, and the order of exposure did not affect the efficacy of the combination (Figure 3c, Supplementary Table v). SAAP-148 at 51.2 µM was similarly effective when applied after and before 70°C NCIH, reducing MSSA by a mean 2.1-log versus 1.5-log CFU/ml, respectively (Figure 3c, Supplementary Table v). In conclusion, sequential exposure of mature biofilm-embedded *S. aureus* to SAAP-148 and NCIH further reduced bacterial counts compared to these treatments alone.

### Effect of NCIH combined with phage ISP on biofilm-embedded *S. aureus*

**Fig. 4:**
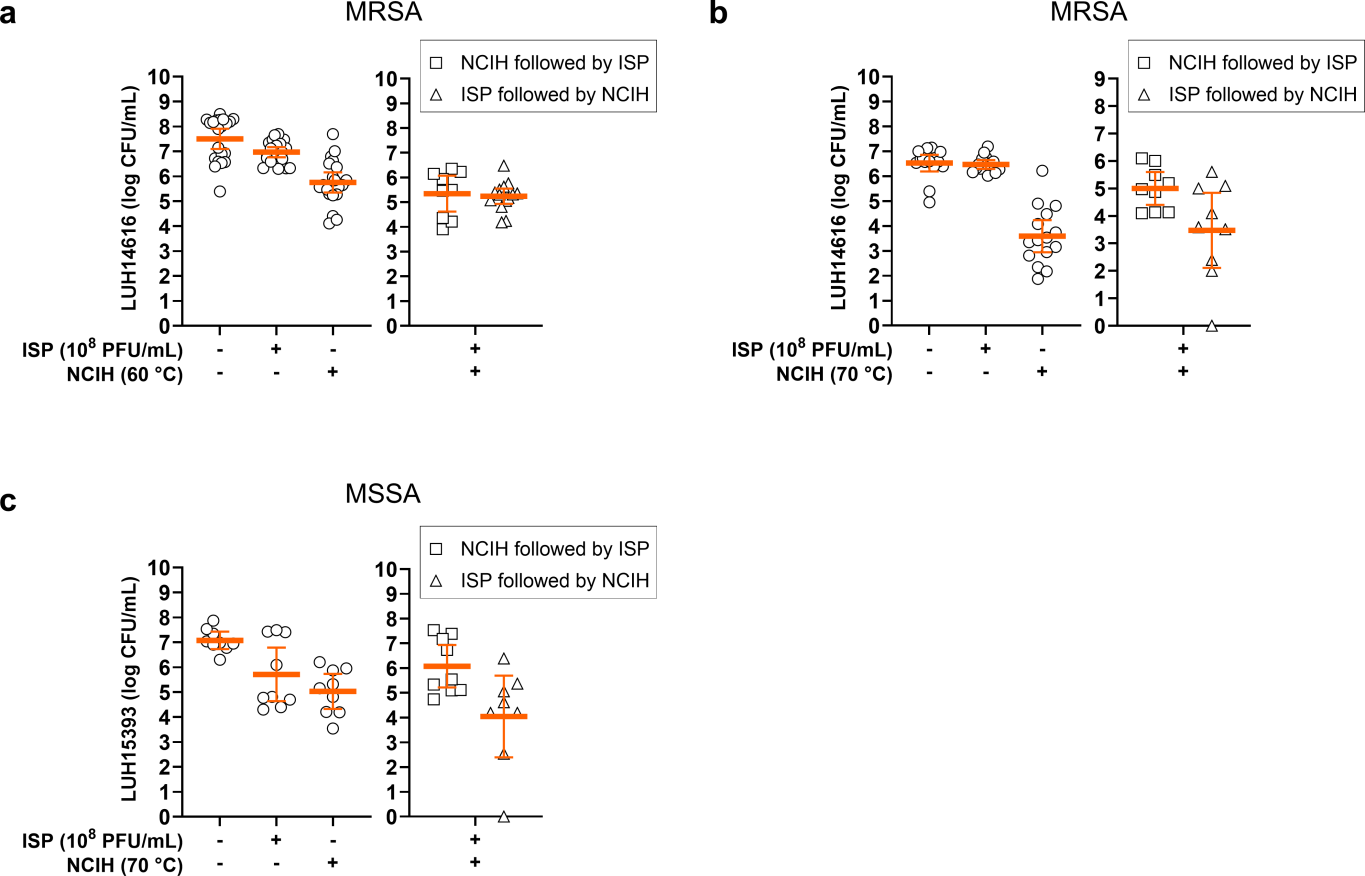
Effect of sequential exposure of *Staphylococcus aureus* biofilms to bacteriophage (phage) ISP and non-contact induction heating (NCIH). Mature biofilms of a) and b) methicillin-resistant *S. aureus* (MRSA) and c) methicillin-sensitive *S. aureus* (MSSA) on titanium-6% aluminium-7% niobium (Ti-6Al-7Nb) discs were sequentially exposed to a) 60°C or b) and c) 70°C NCIH and phage ISP (10^8^ plaque-forming units (PFU)/ml) for 24 hours in brain-heart infusion broth (BHI), or vice versa. The unexposed controls were incubated in BHI. Bacteria were harvested from biofilms by sonication in 0.9% saline with 10 mM ammonium iron (II) sulphate hexahydrate (FAS) to neutralize residual phage activity, and bacterial counts were determined microbiologically (colony-forming units (CFU)/ml). The solid line indicates the mean CFU/ml with the corresponding 95% CIs of the log-transformed data. Results are from ≥ three independent experiments in triplicate or quadruplicate.

Finally, the effect of sequential combinations of NCIH and phage ISP on mature *S. aureus* biofilms was assessed. MRSA within mature biofilms was not additionally reduced by sequential exposure to 60°C or 70°C NCIH and phage ISP at 10^8^ plaque-forming units (PFU)/ml, or vice versa (Figures 4a and 4b). Comparable to the MRSA strain, both orders of sequential exposure to phage ISP and 70°C NCIH did not further reduce biofilm-embedded MSSA (Figure 4c, Supplementary Table vi). Notably, 70°C NCIH before phage exposure resulted in higher bacterial counts of MRSA (mean 1.4-log CFU/ml) and MSSA (mean 1.0-log CFU/ml) biofilms than after exposure to NCIH alone. Thus, phage ISP could not enhance the efficacy of NCIH against biofilm-embedded *S. aureus*.

## Discussion

Antibiotic failure, which is often seen in the treatment of biofilm-associated infections such as FRIs and PJIs, is a serious problem for these patients and poses a significant burden to the healthcare system. Hence, there is an urgent need to develop alternative treatment approaches to combat these bacterial infections. This study determined the effects of NCIH against clinical isolates of *S. aureus* within mature biofilms in an in vitro model using a metal implant mimicking a FRI. Moreover, we assessed the potential of rifampicin/ciprofloxacin, the antimicrobial peptide SAAP-148, and phage ISP to improve the efficacy of NCIH at lower temperatures to combat such biofilms.

In the absence of clinical studies, data from in vitro and in vivo studies have provided some insights into possible safety issues such as thermal necrosis or thermal effects on biomaterials. NCIH to 80°C did not affect the adhesion strength of hydroxyapatite coatings of metal implants, nor the fixation strength of bone cement in an ex vivo study,^27^ illustrating that NCIH up to 80°C will have a low risk of inducing prosthetic implant loosening when fixed within the bone. Although computational models and a small-animal in vivo study showed that induction heating to 75°C was effective against biofilm-embedded bacteria, this resulted in irreversible tissue damage.^28,29^ The safety of NCIH in patients remains to be assessed in future clinical studies, which are currently being initiated.

In line with previous observations, we showed that NCIH as a single approach was highly efficacious in reducing mature biofilm-embedded MSSA and MRSA in a heat-dependent fashion.^8,11^ LIVE/DEAD imaging revealed that NCIH induced bacterial cell death throughout the different biofilm layers, yet NCIH did not cause complete bacterial clearance. Of note, LIVE/DEAD-stained NCIH-exposed biofilms showed clusters of staining at the basal layer, in which some bacterial structures could be identified. An explanation for this phenomenon could be that the NCIH-exposed bacteria released DNA, which clustered and was subsequently stained by the nucleic fluorescent LIVE/DEAD probes.

Since NCIH alone could not induce complete bacterial clearance and might cause irreversible thermal tissue damage, we next investigated whether antibiotics, SAAP-148, and phage ISP could enhance the efficacy of NCIH at lower temperatures (60°C and 70°C). Simultaneous exposure of these agents to such temperatures would likely affect their stability and activity. Hence, we sequentially exposed the biofilm-embedded bacteria to these combinatory approaches, which would also indicate which potential application would be most potent in clinical cases of FRI.

First, we tested the ability of antibiotics to enhance the efficacy of NCIH in reducing biofilm-embedded *S. aureus*. Previously, NCIH effectively reduced biofilm-embedded *S. aureus*, *S. epidermidis,* and *P. aeruginosa*, and synergized with antibiotics in reducing the bacterial burden within such biofilms.^12-14,29^ Here, we focused on rifampicin/ciprofloxacin due to rifampicin’s biofilm-penetrating activity and the recommended use of rifampicin-based treatment following surgical treatment against staphylococcal implant infections.^30^ Rifampicin/ciprofloxacin followed by NCIH effectively reduced MRSA and MSSA within biofilms in a dose-dependent manner. Our results showed that the exposure of biofilm-embedded *S. aureus* to rifampicin/ciprofloxacin for 24 hours, and subsequently to NCIH, was more effective than the other way around. It is possible that antibiotics injured the surviving bacterial fraction, and subsequent NCIH exposure resulted in the final push towards bacterial cell death. The mechanism behind this phenomenon remains to be elucidated. Nevertheless, for the rifampicin/ciprofloxacin-sensitive MSSA strain, the influence of exposure order was reduced when we applied a high antibiotic dose (256 × MBC rifampicin). These results indicate that the order of exposure of different antimicrobial methods has less influence on treatment efficacy, as long as the second exposure is sufficient to eliminate bacteria or hamper bacterial outgrowth. In line with this, previous research showed that seven-day mature MSSA biofilms cultured on Ti-6Al-4V coupons were synergistically reduced by NCIH followed by rifampicin/ciprofloxacin at relatively high concentrations.^14^ Despite the differences in experimental set-up between these studies (e.g. bacterial strains, NCIH device, bacterial load within biofilm), we speculate that the composition of the metal implant material does not affect the efficacy of NCIH and antibiotics against *S. aureus* biofilms. Furthermore, the ciprofloxacin concentrations used in our study were high for the rifampicin/ciprofloxacin-resistant MRSA and did not represent clinically reachable concentrations. Nevertheless, exposure of such biofilms to clinically relevant concentrations of rifampicin/ciprofloxacin followed by 70°C NCIH yielded similar synergistic reductions. This illustrates that the exposure to antibiotics, even if bacteria resist them, can increase the bacterial susceptibility to NCIH. We speculate that this phenomenon is caused by collateral sensitivity,^31^ i.e. antibiotic-resistant bacteria show increased sensitivity to heat exposure. This hypothesis is supported by the observation that the MRSA strain was more susceptible to NCIH ranging from 60°C to 70°C as a single approach than the MSSA strain.

Next, we investigated the ability of SAAP-148 to enhance the efficacy of NCIH on biofilm-embedded *S. aureus*. Indeed, SAAP-148 enhanced the efficacy of NCIH for both sequential treatment orders against MSSA and MRSA biofilms. Importantly, it should be mentioned that the experimental set-up of the SAAP-148 exposure differed from that of rifampicin/ciprofloxacin and phage ISP due to SAAP-148’s relatively short activity and stability. Bacteria were exposed to SAAP-148 for two hours, while exposure to antibiotics and phage ISP occurred for 24 hours. Since the activity of SAAP-148 is affected by BHI, it was dissolved in PBS, while rifampicin/ciprofloxacin and phage ISP were dissolved in BHI. Nutrient-rich media like BHI and incubation time are important factors for bacterial replication, which is reflected by the difference in bacterial load of the unexposed controls, i.e. 8-log versus 7-log CFU/ml for 24 hours’ incubation in BHI and two hours’ incubation in PBS, respectively. These differences should be considered when comparing the results of the various treatment combinations to prevent overestimating the effect of SAAP-148. Further, SAAP-148 as a single approach appeared more effective against the biofilm-embedded MRSA than the MSSA strain, while these bacteria in planktonic phase showed similar susceptibilities. It remains to be evaluated whether the biofilm-matrix properties of these strains differ, and whether this influenced the penetration and/or efficacy of SAAP-148.

Lastly, we assessed the ability of phage ISP to improve the efficacy of NCIH against biofilm-embedded *S. aureus*. Previously, phage ISP as a single agent was not effective against biofilm-embedded MRSA and MSSA on Ti-6Al-7Nb discs.^18^ Our results showed that exposure to phage ISP prior to and following NCIH exposure did not further reduce bacterial counts. Instead, subjecting MRSA and MSSA biofilms to phage ISP and 70°C NCIH resulted in higher bacterial counts than the treatments alone. Thus, phage ISP exposure did not further sensitize the phage-tolerant biofilm-embedded bacteria to NCIH or vice versa. Instead, NCIH might affect the bacterial cell wall as previously suggested,^13^ thereby impairing phage adsorption to the bacterial cell and hampering its efficacy.

Even the most effective combination, i.e. antibiotics followed by NCIH, did not completely eradicate all bacteria within biofilms. Hence, the possibility that the remaining bacterial population can be cleared by host immune cells, prolonged antibiotics exposure, or an additional heat shock should be considered. The current treatment of these infections consists of surgical interventions and antibiotics, or even implant revision or removal.^32^ Based on our data, we propose that exposure to NCIH would be most effective when antibiotic treatment has already started. Applying this approach could potentially reduce cases of implant revision, a more invasive and costly procedure.

Our study suffers from some limitations. First of all, we did not incorporate the host’s immune cells in our experiments. It is crucial to conduct future studies to determine the influence of the host’s immune system on clearing the remaining bacterial load after antibiotics followed by NCIH, as well as the safety and efficacy of these approaches in vivo. Second, mechanical cleaning was not incorporated into our experimental setup. Previous research, however, showed that the additive effect of mechanical cleaning was limited in combination with antibiotics and NCIH against *S. aureus* biofilms.^14^ Third, the 95% CIs indicate high variation for some conditions, requiring some caution when interpreting the results. This variation was observed within, as well as between, independent experiments. Further, only one type of antibiotic combination, antimicrobial peptide, and phage was used in this study. It is possible that other antimicrobial agents with different mechanisms of action might behave differently when sequentially combined with NCIH. For example, phages with more potent antibiofilm activities, such as Sb-1,^33^ might further improve the efficacy of NCIH against biofilm-embedded *S. aureus*.

To conclude, this study has demonstrated that NCIH as a single approach was highly effective in diminishing mature biofilm-embedded MRSA and MSSA. Moreover, rifampicin/ciprofloxacin and SAAP-148, but not phage ISP, further enhanced the efficacy of NCIH at lower temperatures against biofilm-embedded *S. aureus* compared to when those treatments were applied alone. Our data indicate that NCIH was more effective when applied after than prior to antibiotic exposure at suboptimal concentrations, suggesting that this approach could be most suitable in a clinical setting when antibiotic treatment has already started.

## Data Availability

The data that support the findings for this study are available to other researchers from the corresponding author upon reasonable request.
